# Translation directionality and translator anxiety: Evidence from eye movements in L1-L2 translation

**DOI:** 10.3389/fpsyg.2023.1120140

**Published:** 2023-02-21

**Authors:** Juan Jia, Ziyu Wei, Heben Cheng, Xiaolu Wang

**Affiliations:** ^1^School of Foreign Languages, Hangzhou City University, Hangzhou, China; ^2^Hangzhou Guancheng Wulin Middle School, Hangzhou, China; ^3^School of International Studies, Zhejiang University, Hangzhou, China; ^4^School of Humanities and Communication Arts, Western Sydney University, Penrith, NSW, Australia

**Keywords:** translation directionality, translator anxiety, cognitive load, beck anxiety inventory, eye-tracking

## Abstract

While considerable research on the impact of anxiety on second language learning has been carried out in international contexts, the impact of anxiety on the translator’s undertaking L2 translation, a sort of anxiety arising from the translation directionality, as well as the structure of cognitive mechanism for translational anxiety, remain under-explored. Adopting the eye-tracking and key-logging approach to data collection, this study implemented an eye-tracking experiment with EFL learners at a Chinese university to probe into how the participants responded to L1 and L2 translation-tasks and the mechanism involved in these processes. It is found that translation directionality does have a great impact on the processing of translation, which causes the change of cognitive load and then leads to the change of levels in translator anxiety. The finding further confirms the key premises of the Processing Proficiency Model and the Revised Hierarchical Model with attendant implications for translation processes.

## Introduction

Second or foreign language learning is affected by a wide range of factors, including ‘cognitive factors (language aptitude, learning strategies), affective factors (attitudes, motivation, and anxiety), metacognitive factors, and demographic factors (Henter, 2014, p. 373). Amongst these factors, anxiety is one of the most notable variables of influence since researchers such as Horwitz et al. (1986) and MacIntyre and Gardner (1994) demonstrated that the anxiety experienced by foreign language learners is distinctive in nature. MacIntyre and Gardner (1994, p. 284) defined anxiety as “a feeling of tension and apprehension specifically associated with second language contexts, including speaking, listening, and learning.” Multiple studies (e.g., Horwitz et al., 1986; Phillips, 1992; Woodrow, 2006) investigating the phenomenon of language learning anxiety suggested that anxiety and foreign language performance are negatively related. While many studies have examined the effect of L2 anxiety on L2 learners’ performance or achievement in class, there is limited research on the impact of anxiety on learners within translation-training settings (Yan and Wang, 2012) and even less so on the cognitive mechanism whereby anxiety influences cognitive operations within the process of translation. Therefore, the relationship between translation directionality and translator anxiety and its cognitive mechanism become the key foci of the current paper.

With reference to the cognitive mechanism involved in translation, the general theory of anxiety efficacy processing efficiency theory (PET) advanced by Eysenck and Calvo (1992) is of considerable importance as it helps to explore the influence of anxiety on cognitive process which in turn affects individual’s task performance. Earlier studies on translation direction have tended to focus on output quality in different translation directions. Kroll and Stewart (1994) revised hierarchical model (RHM) is of great help for a better understanding of the process of translation from the perspective of cognition as the model describes the bilinguals’ dynamic development of the conceptual representation of second language vocabulary along with the improvement in second language proficiency. The value of this model is that it provides a basis for the study of translation from a cognitive perspective. Research on translation process tends to focus on the influence of anxiety level on cognitive load, the influence of translation direction on cognitive load, and the influence of translation direction, anxiety, and cognitive load on translation performance. However, the correlation between cognitive load and anxiety related to translation direction is under-explored, particularly in relation to the possible differences of cognitive load in the source text (ST) and the target text (TT) mediating different translation directions. Thus the present study is hypothesized that L2 translation direction is likely to arouse more translation anxiety than L1 translation as anxiety tends to weaken the individual’s processing efficiency with more cognitive efforts used, and while cognitive efforts can be measured by the translator’s outward performance with the tools of key logging and eye movements, anxiety can be measured by the beck anxiety inventory (BAI) after the immediate task performance. Our study, in this way, was designed not only to address the gaps highlighted in the following research questions but also to examine the cognitive mechanism of translation anxiety in different translation directions.

1. In the process of translation, what relationship can be found between the translators’ foreign language anxiety (FLA) and their cognitive load?
2. In L1 translation and L2 translation, which translation direction is likely to arouse more translation anxiety, and why?
3. In what way is the cognitive mechanism of translation anxiety distinguishable between L1 and L2 translations?

## Key terms and previous studies

### Foreign language anxiety

Synchronically, studies on language acquisition, especially second or foreign language learning, were conducted from cognitive factors to affective factors. Among them, the affective filter hypothesis (Krashen, 1985) is of pivotal importance to the present study as it relates to the role of affective variables (e.g., anxiety). According to Krashen (1985), the affective filter is a mental block which obstructs language learners from fully utilizing the comprehensible input they receive in the process of acquiring a language. The affective filter does not restrain acquisition directly, but it can prevent input from reaching the part of the brain responsible for language acquisition. Drawing upon Krashen (1982) and Hammond (1990, p. 65), observed that “the affective variables of motivation, self-confidence and anxiety” exercise a deep influence on the acquisition of language. Oxford (1999) contended that “anxiety” is the first and most factor of the affective variables affecting language acquisition.

As translation activities must be generated in a foreign language environment, foreign language anxiety produced in such environment affects translation behavior. With reference to the cognitive mechanism involved in translation, PET, the general theory of anxiety efficacy suggests that individuals with high anxiety tend to pay more attention to their performance and evaluation by others, which gives rise to their negative thoughts in mind. Given that these negative thoughts occupy large parts of the working memory resources in central executive system and/or the auditory rehearsal loop, their effect on performance in the form of anxiety are very significant. Task difficulty also impacts the performance of individuals experiencing anxiety. As the level of task difficulty is determined by the resource requirements of central execution system and auditory rehearsal loop, the higher the requirements, the more difficult the task is. Multiple task completion/experiment-based studies have provided support for the credibility of this theory (Dornic, 1977, 1980; Weinberg, 1978; Eysenck, 1985, etc). In the Chinese context, anxiety has been accepted as a negative correlation with translation (Zheng, 2003). Research in the Chinese context has also examined the influence of anxiety on language cognitive processing, focusing on the negative effect of trait anxiety on emotional word processing by eye-tracking (Xu, 2014), and offering evidence in support of PET assumptions (Lin, 2004; Yu, 2019).

### Translation directionality

An important factor affecting FLA is translation directionality. Translation directionality had long been completely rejected or neglected in traditional translation studies until the call appeared by various scholars in 1990s. Pavlović (2007, p. 80) described “directionality” as whether the translation is done into individuals’ mother tongue/language of habitual use, or into their second language/foreign language. The traditional view regarded translation directionality as a combination of dismissing L2 translation and taking LI translation as “the unwritten rule” (Pokorn, 2000). Now the consensus has been reached that “directionality” can be done from a foreign language to a mother tongue or vice versa in the translation process (Beeby, 2009). As terms “mother tongue” and “foreign language” are seen as problematic due to feeling or ideology “colored” (Pedersen, 2000), the neutral description “first language -L1” and “the second language-L2” are adopted for this work. Thus, translation directionality refers to the direction of translation from L2 to L1 (L1 translation) or from L1 to L2 (L2 translation).

Previous studies on translation directionality have been limited to theoretical discussions on the quality of products in two opposite translation directions (Kroll and Stewart, 1994). They showed that translators encounter similar problems in the process of translation while the quality of the final products of translation depends on the direction of translation. To be specific, the quality of L1 translation turned out to be much higher than that of L2 translation. The aspects which contributed to the direction difference can be assigned as translators’ translation competence, personal preferences, text types, environmental conditions, etc (Pavlović, 2007). Actually, it is the translator’s cognitive processes and affective factors (anxiety) in different translation directions determine the quality of the final products of translation.

### Empirical studies on translation directionality

Most previous studies on the relationship between cognitive load of translation and translation directionality have been premised on behavioral evidence (Harley, 2001). The proposed model, RHM, specifically figures out “translation asymmetry” in translation activities, which means translating/interpreting a word into a second language requires more cognitive effort than undertaking this in the first language (Kroll and Stewart, 1994). Since the mid-to-late 1980s, evolving science, technology and methodological innovation have opened up avenues for the generation and use of objective data to describe translation phenomena and demonstrate theoretical hypotheses. A breakthrough in this field was the introduction of the Think-alound Protocols (TAPs) in the field of psychology. Inspired by this, researchers concerning translation process began to draw upon other disciplinary methods, such as key-logging, screen recording, webcam recording, eye-tracking and neuroscience methods which effectively supplemented or replaced the traditional TAPs. However, the application of key-logging and/or eye-tracking technology to research on directionality has still been limited in translation (Ferreira, 2014; de Lima Fonseca, 2015) and interpreting research.

The traditional view of directionality which is based on assumptions rather than on empirical data has been challenged by international research. Investigating cognitive effort in translation processes by deploying the eye-tracking experiment with student and professional subjects in translation tasks, Jensen and Pavlović (2009) found that the cognitive effort required in the TT processing is significantly higher than in the ST processing in both translation directions. Chinese scholars have also explored the correlation between translation directionality and cognitive load by key-logging and/or eye-tracking. Deploying eye-tracking and fMRI, Chang (2009) explored the validity of RHM at textual level with physiological and neurological data on cognitive loading of 16 participants (Chinese as their first language and English as their foreign language). Based on Chang’s work, Feng (2017) sought to verify three hypotheses by collecting eye-tracking data pertaining to task time, pupil diameter, average fixation time, total fixation time and fixation frequency of 20 student translators, concluding that while the cognitive load in L2 translation is higher than that in L1 translation, the relationship between cognitive load and text type (ST and TT) cannot be conclusively established. Wang (2019) also explored the effects of translation directionality and text difficulty on cognitive load and translation performance of 16 Chinese students majoring in translation and interpretation by means of key-logging, eye-tracking and questionnaires and found that with STs of low difficulty, cognitive load in Chinese-English translation is higher than English-Chinese translation; with STs of high difficulty, the cognitive load in Chinese-English translation is not necessarily higher than English-Chinese translation.

To sum up, the above discussion shows that the proposed PET offers a new perspective in translation process research (TPR) and that the hypothesized effects of anxiety on cognitive load and performance have been well-tested. However, the relationship between anxiety and cognitive load has only been confirmed in one direction, while the effects of cognitive load level on anxiety level remain under-researched. Further, while most studies have confirmed the relationship between translation direction and cognitive load, research questions related to translation direction, such as whether there is a significant difference of cognitive load in the ST and the TT between different translation directions, still remain to be investigated.

## Methods

### Translation anxiety experiment

In order to explore the correlations and differences of the following factors, i.e., EFL learners’ cognitive load, translation directionality and anxiety between L1 translation and L2 translation, first, participants were selected randomly to two groups. Then, they were required to complete a pre-test to ensure similar level of English proficiency. Next, as they performed E-C and C-E translation tasks, their anxiety was compared by means of their eye-movement and keystroke data.

### Eye-tracking

Eye-tracking technology which is the measurement of eye activities is widely used in TPR, especially in cognitive translation research (CTS). Eye-tracking collects eye data by using a computer-connected device called “eye-tracker” which could either be remote or head-mounted. In the present study, we tracked the eye movement data only from the participant’s right eye by the SR Research Eyelink 1,000 plus system at a sampling rate of 1,000 Hz. The experiment was carried out on Dell P1917S with a 19-inch monitor, which has a refresh rate of 75 Hz and a screen resolution of 1024*768 pixels. A chin rest with forehead support was used in the experiment in order to minimize the interference caused by participants’ head movements.

### Translog II

In the present study, Translog II is used to record the user activity data including all the gaze movements and keystrokes which is connected to an SR Research Eyelink 1,000 plus system and a specific key-logging software. Translog II collects eye movements data in the form of gaze-sample points and fixations. In order to track gaze-sample points and fixations of the participants effectively, STs were set on the left window and TTs were on the right window in “Configure Experiment” step in Translog II Supervisor. It also collects keystroke data through the specific key-logging software installed on the subject’s computer by recording his/her keyboard activities. Information includes keystroke and mouse movements data such as deletions, insertions, corrections, editorial changes together with total task duration and time intervals between keystrokes. According to the distribution of keyboard activities, the translation process consists of initial orientation phase, middle drafting phase and revision and monitoring phase (Feng and Wang, 2016). Translog II finally creates a log files which contains all the data mentioned above when the user finishes their tasks of reading, writing and translating a text.

### Beck anxiety inventory

In this study, the beck anxiety inventory (BAI) was used to test the anxiety level of participants during L2-L1 (English-Chinese, E-C) or L1-L2 (Chinese-English, C-E) translation. Beck anxiety inventory was created by Aaron T. Beck et al., and it comprises a self-report inventory consisting of 21 multiple-choice items describing emotional, physiological, and cognitive symptoms of anxiety.

### Participants

A total of 78 undergraduate and postgraduate students [55 females, 23 males; mean age = 22.1, standard deviation (SD) = 2.58] from a top university in China, participated in the experiment. To maintain the integrity of the experiment, appropriate selection was ensured to exclude the participants with prior translation experience and English-major background. All of the participants were right-handed and had Mandarin Chinese as their L1 and English as their L2. They also had normal or correct-to-normal vision. None of them had any history of neurological or psychiatric disorders. Before the experiment, all the participants signed an ethical statement describing the ethical guidelines under which this research was carried out and were paid a small amount after the experiment.

### Materials

In the pre-test, translation texts were carefully selected from CET-4 simulation test in order to suit subjects’ language ability and avoid accidentally translating before (see Supplementary Appendix S1). During the preparation for the experimental materials, the difficulty of formal texts were carefully controlled to correspond to the learners’ level of knowledge. The texts of the formal experiment are also divided into Chinese text and English text. Different from the pre-test texts, the formal texts were a Chinese-English translation test and its reference answer selected from CET-4 simulation test. The Chinese text was used as the experimental material of C-E translation, and the English version of the reference answer was used as the experimental materials of E-C translation (see Supplementary Appendix S2). Time for the translation task was limited within 30 min adhering to the CET-4 rules.

### Procedures

At the recruitment stage, the participants completed the questionnaires on their personal background and their score of CET-4^1^ as well as their score of translation in CET-4. In order to ensure the participants with similar English proficiency level, a pre-test was conducted with suitable translation tasks and the participants with total scores above 18 points were enrolled. In the formal experiment, after each participant was informed of the test steps, the subjects were positioned at their stations in accordance with the necessary guidelines to ensure that the experiment was not adversely impacted by the physical movement of the participants. During the experiment, the participants did the translation task and output the translation in the right window of Translog II User. And the monocular mode of the SR Research Eyelink 1,000 plus system was used in this study, so only the right eye of each participants was tracked. After completing the translation task, the participants immediately filled out a BAI scale based on their personal physiological symptoms and evaluations. During the experiment, the participants were not aware that anxiety evaluation would be provided after the translation tasks, otherwise, they would not totally focus their attention on the translation task. Moreover, if they had known that they would evaluate their anxiety after the translation task, they might think of some strategy to show their confidence. If that happened, we would never collect the true data for their anxiety.

## Results and analysis

The data collected from Translog II software and SR Research Eyelink 1,000 plus system mainly include the task duration, fixation count (displayed, respectively, on ST and TT), fixation duration, pause count, and pause duration of the 68 participants from the two groups. The anxiety level was obtained from their BAI scores. All of the data were analyzed in SPSS 20.0. In this study, most of the key-logging and eye-tracking data in this experiment were coded and could not be analyzed directly. Therefore, data analyzing tools adopted for this study included CRITT TPR-DB and SPSS 20.0. CRITT is the abbreviation of Center for Research and Innovation in Translation and Translation Technology. The tables selected for the follow-up data processing in this study include the session summary table, the fixation data summary table and the keystroke data summary table.

### Anxiety difference analysis

In this part, the difference between the cognitive load and the BAI scores of E-C and C-E tasks was tested by independent-samples *t*-test. What is more, as the variables of fixation count and reading time were displayed, respectively, in ST and TT in the tables generated from CRITT TPR-DB, the difference of cognitive load between ST and TT processings was also tested by independent-samples *t*-test.

### Descriptive results

**Figure 1:**
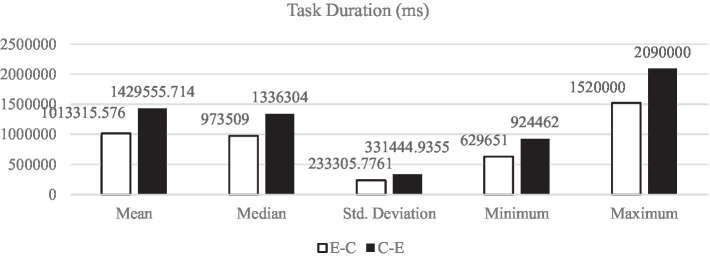
Descriptive results of TD in L1 and L2 translations.

**Figure 2:**
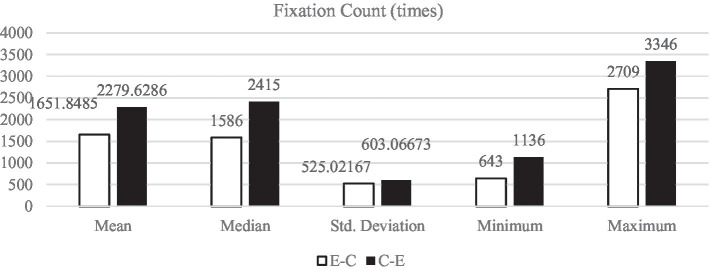
Descriptive results of FC in L1 and L2 translations.

**Figure 3:**
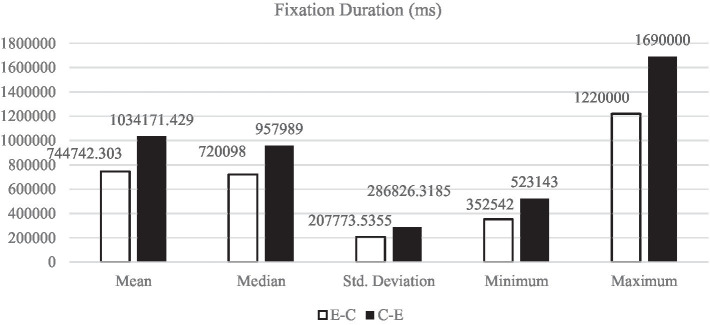
Descriptive results of FD in L1 and L2 translations.

**Figure 4:**
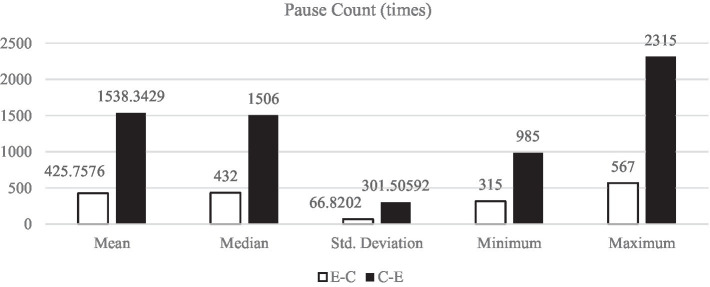
Descriptive results of PC in L1 and L2 translations.

**Figure 5:**
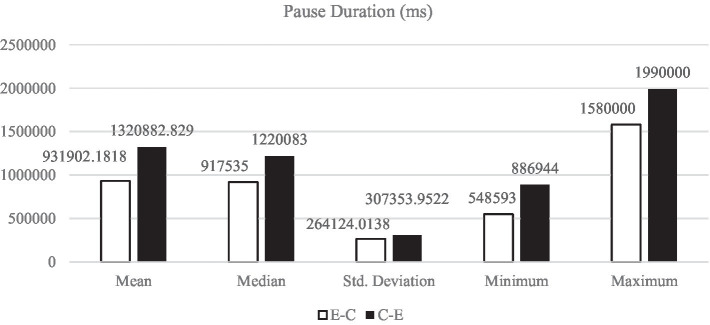
Descriptive results of PD in L1 and L2 translations.

**Figure 6:**
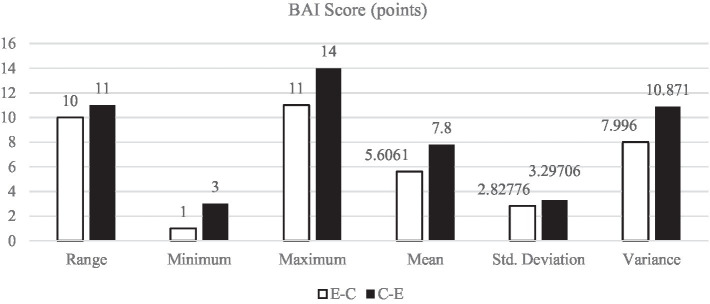
Descriptive results of BAI score in L1 and L2 translations.

Figures 1–5 display the descriptive results of key-logging and eye-tracking data of the two groups (E-C group and C-E group) respectively. And Figure 6 displays the descriptive results of BAI scores of the two groups.

#### Task duration

TD refers to the production duration of a final TT per session. It starts from the beginning of the session recording until it is stopped. Estimating how long a task will take to complete (i.e., the task duration) is important for our research, for the data regarding TD indicate that the more time used for a task completion, the more cognitive efforts are used in the performance. As we can see from Figure 1, all the descriptive results of TD in E-C translation (*M*_E-C_ = 1013315.58 ms, *SD*_E-C_ = 233305.78 ms) are lower than those in C-E translation (*M*_C-E_ = 1429555.71 ms, *SD*_C-E_ = 331444.94 ms).

#### Fixation count

Fixation count refers to the total number of fixations on the source and TTs during the presentation of stimulus materials. The cumulative result of each saccade is the total number of fixations. In general, the more times the subjects focus on a certain area, the greater attention of this area is paid by the translator. Figure 2 shows that all the descriptive results of FC in E-C translation (*M*_E-C_ = 1651.85 times, *SD*_E-C_ = 525.02 times) are lower than those in C-E translation (*M*_C-E_ = 2279.63 times, *SD*_C-E_ = 603.07 times).

#### Fixation duration

Fixation duration stands for the duration of each fixation. Generally speaking, the longer the subject looks at a certain area, the more interested he/she is in the area. In translation process, it may also indicate that the participant is more confused about the contents of the materials, and vice versa. Figure 3 shows that all the descriptive results of FD in E-C translation (*M*_E-C_ = 744742.30 ms, *SD*_E-C_ = 207773.54 ms) are lower than those in C-E translation (*M*_C-E_ = 1034171.43 ms, *SD*_C-E_ = 286826.32 ms).

#### Pause count

Pause count refers to the total number of pauses when the participants produce their TTs. It is agreed that the more pause count, the more cognitive load and less cognitive resources in the translation process, and vice versa. All the descriptive results of PC in E-C translation (*M*_E-C_ = 425.76 times, *SD*_E-C_ = 66.82 times) are much lower than those in C-E translation (*M*_C-E_ = 1538.34 times, *SD*_C-E_ = 301.51 times).

#### Pause duration

Pause duration refers to typing pause duration prior to a keystroke. According to Muñoz Martín and Apfelthaler (2021), 200 ms can capture“all translators’ disfluencies; typing goal breaks and changes; reactions to visual stimuli; and the interaction of cognitive, perceptual, and action operations (i.e., embodiment) (p. 24).” The average fixation duration and planning of motor saccades in the present study is 200 ms. In our research the pause duration is longer, the more cognitive load is carried by the participant, and vice versa. Based on the results of Figure 5, all the descriptive results of PD in E-C translation (*M*_E-C_ = 931902.18 ms, *SD*_E-C_ = 264124.01 ms) are lower than those in C-E translation (*M*_C-E_ = 1320882.83 ms, *SD*_C-E_ = 307353.95 ms).

The descriptive results of BAI score are the same with those of the five variables mentioned above. All the descriptive results of BAI score in E-C translation (*M*_E-C_ = 5.61 points, *SD*_E-C_ = 2.83 points) are lower than those in C-E translation (*M*_C-E_ = 7.8 points, *SD*_C-E_ = 3.30 points).

### Test of normality

Before independent-samples *t*-test, it is necessary to make sure whether the data is normal distribution. Therefore, the tests of normality for five variables are examined. Tables 1, 2 are the tests of normality about the key-logging, eye-tracking and BAI score data.

**Table 1:** Tests of normality of key-logging and eye-tracking data in L1 and L2 translation.

L1 and L2	Kolmogorov-Smirnov			Shapiro–Wilk		
	Statistic	df	Sig.	Statistic	df	Sig.
Dur	0.100	33	0.200	0.974	33	0.600
	0.138	35	0.089	0.944	35	0.076
FC	0.123	33	0.200	0.966	33	0.367
	0.114	35	0.200	0.959	35	0.216
FD	0.130	33	0.171	0.948	33	0.115
	0.146	35	0.058	0.958	35	0.198
PC	0.103	33	0.200	0.963	33	0.318
	0.101	35	0.200	0.962	35	0.260
PD	0.117	33	0.200	0.946	33	0.099
	0.154	35	0.034	0.948	35	0.102

**Table 2:** Tests of normality of BAI scores in L1 and L2 translations.

BAI	Kolmogorov-Smirnov			Shapiro–Wilk		
	Statistic	df	Sig.	Statistic	df	Sig.
E-C	0.135	33	0.135	0.943	33	0.083
C-E	0.128	33	0.185	0.939	33	0.062

Tables 1, 2 show the tests of normality for the results of the task duration (TD), fixation count (FC), fixation duration (FD), pause count (PC), pause duration (PD), and BAI scores, which help determine exactly whether the distribution is normal or not. In these tables, the significant values of both Kolmogorov–Smirnov and Shapiro–Wilk are displayed. According to the results of the tables, the significant values of the five variables of key-logging and eye-tracking and BAI scores from both groups are larger than 0.05, which means that the key-logging and eye-tracking data and BAI scores of the two groups are in accordance with normal distribution, so significant difference analysis can be conducted in the next step.

### Difference of cognitive load between L1 and L2 translations

After the tests of normality, the difference between key-logging and eye-tracking data of the two groups is tested by independent-samples *t*-test. The results are presented in Table 3.

**Table 3:** Independent sample test of key-logging and eye-tracking data in L1 and L2 translations.

	Levene’s test for equality of variances	*t*-test for equality of means					
	*F*	Sig.	*t*	df	Sig. (two-tailed)	Mean difference	Standard error difference
Dur.	8.592	0.005	−5.955	66	0.000	−416240.1	69897.1
			−6.015	61.174	0.000	−416240.1	69196.66
FC	1.046	0.31	−4.567	66	0.000	−627.7801	137.4731
			−4.585	65.596	0.000	−627.7801	136.9091
FD	7.619	0.007	−4.741	66	0.000	−289429.1	61052.93
			−4.785	61.979	0.000	−289429.1	60487.43
PC	33.306	0.000	−20.715	66	0.000	−1112.585	53.70809
			−21.284	37.526	0.000	−1112.585	52.27438
PD	2.283	0.136	−5.582	66	0.000	−388980.6	69688.26
			−5.607	65.454	0.000	−388980.6	69375.98

The results of Table 3 shows that there is a significant difference of task duration between E-C task (1013315.58 ± 233305.78) and C-E task (1429555.71 ± 331444.94), *t*(61.174) = −6.015, *p* < 0.001. As for fixation count, according to the above data, there is a significant difference of fixation count between E-C task (1651.85 ± 525.02) and C-E task (2279.63 ± 603.07), *t*(66) = −4.567, *p* < 0.001. Significant difference of fixation duration between E-C task (744742.30 ± 207773.54) and C-E task (1034171.43 ± 286826.32) is detected in the tables, *t*(61.979) = −4.785, *p* < 0.001. There also exits a significant difference of pause count between E-C task (425.76 ± 66.82) and C-E task (1538.34 ± 301.51), *t*(37.526) = −21.284, *p* < 0.001, and the pause duration in E-C task (931902.18 ± 264124.01) is significantly lower than that in C-E task (1320882.83 ± 307353.95), *t*(66) = −5.582, *p* < 0.001.

In order to explore the reason of significantly higher cognitive load in L2 translation, this part mainly displays the results of the difference of cognitive load in ST and TT between two translation directions. Independent-samples *t*-test is used to analyze the fixation count on source text (FCS), fixation count on target test (FCT), reading time on source text (RTS) and reading time on target text (RTT) in both L1 and L2 translations, aiming to explore whether there is a significant difference of cognitive load in the ST and the TT between different translation directions. The results are presented in Tables 4, 5.

**Table 4:** Group statistics of key-logging and eye-tracking data in ST and TT processings.

	Direction	*N*	Mean	Standard deviation	Standard error mean
FCS	E-C	33	926.2727	319.30582	55.58401
	C-E	35	813.5714	271.86332	45.95329
RTS	E-C	33	396157.0303	111004.93957	19323.47968
	C-E	35	348357.3714	108085.29645	18269.74963
FCT	E-C	33	725.5758	292.63939	50.94198
	C-E	35	1153.0000	434.97153	73.52361
RTT	E-C	33	330100.4242	154493.79791	26893.91820
	C-E	35	641242.6286	233606.78691	39486.75398

**Table 5:** Independent sample test of key-logging and eye-tracking data in ST and TT processings.

	Levene’s test for equality of variances		*t*-test for equality of means				
	*F*	Sig.	*t*	df	Sig. (two-tailed)	Mean difference	Standard error difference
FCS	0.165	0.686	1.57	66	0.121	112.7013	71.77733
			1.563	62.995	0.123	112.7013	72.11995
RTS	0.043	0.837	1.799	66	0.077	47799.66	26571.72
			1.797	65.511	0.077	47799.66	26592.87
FCT	5.215	0.026	−4.725	66	0.000	−427.4242	90.45912
			−4.779	59.83	0.000	−427.4242	89.44723
RTT	6.684	0.012	−6.437	66	0.000	−311142.2	48336.93
			−6.513	59.302	0.000	−311142.2	47775.38

The results of Tables 4, 5 show that there exists a difference of fixation count on the ST between E-C translation (926.27 ± 319.31) and C-E translation (813.57 ± 271.86), but such difference is not significant, *t*(66) = 1.57, *p* = 0.121.

Also, no significant difference of reading time on the ST between E-C translation (396157.03 ± 111004.94) and C-E translation (348357.37 ± 108085.30) is detected in this study, *t*(66) = 1.799, *p* = 0.077. However, there is a significant difference of fixation count on the TT between E-C translation (725.58 ± 292.64) and C-E translation (1153.00 ± 434.97), *t*(59.83) = −4.779, *p* < 0.001. Also, there is a significant difference of reading time on the TT between E-C translation (330100.42 ± 154493.80) and C-E translation (641242.63 ± 233606.79), t(59.302) = −6.513, *p* < 0.001.

### Difference of beck anxiety inventory score between L1 and L2 translations

The results of BAI scores are presented in Table 6.

**Table 6:** Independent sample test of BAI scores.

	Levene’s test for equality of variances		*t*-test for equality of means				
	*F*	Sig.	*t*	df	Sig. (two-tailed)	Mean difference	Standard error difference
							
BAI	0.462	0.499	−2.937	66	0.005	−2.19394	0.74696
			−2.951	65.431	0.004	−2.19394	0.74357

The results of independent-samples *t*-test in the table above show that there is a significant difference of BAI scores between E-C task (5.61 ± 2.83) and C-E task (7.80 ± 3.30), *t*(66) = −2.937, *p* < 0.05.

### Correlation analysis between cognitive load and anxiety

This part focuses on the correlation between key-logging and eye-tracking data and BAI scores of the two groups. As the normal distribution of the data of the study has been verified in the part of difference analysis, the results of Pearson Product–Moment correlation are displayed in Table 7.

**Table 7:** Correlations between variables in L1 and L2 translation.

	Dur.	FC	FD	PC	PD	BAI	
L1—BAI	Pearson correlation	0.928	0.611	0.814	0.481	0.926	1
	Sig. (two-tailed)	0.000	0.000	0.000	0.005	0.000	
	*N*	33	33	33	33	33	33
L2—BAI	Pearson correlation	0.962	0.807	0.916	0.329	0.951	1
	Sig. (two-tailed)	0.000	0.000	0.000	0.053	0.000	
	*N*	35	35	35	35	35	35

The results of Pearson Product–Moment correlation test in the tables above show that there is a significant and positive correlation between BAI scores and task duration in both E-C (*r* = 0.928, *p* < 0.01) and C-E tasks (*r* = 0.962, *p* < 0.01) and also between BAI scores and fixation count in both E-C (*r* = 0.611, *p* < 0.01) and C-E tasks (*r* = 0.807, *p* < 0.01). As for the relationship between BAI scores and fixation duration, there is a significant and positive correlation in both E-C (*r* = 0.814, *p* < 0.01) and C-E tasks (*r* = 0.916, *p* < 0.01). A positive correlation between BAI scores and pause count in both E-C (*r* = 0.481, *p* < 0.01) and C-E tasks (*r* = 0.329, *p* = 0.053) is also detected in the experiment, but the correlation in the C-E task is less significant than that in the E-C task. Besides, there is a significant and positive correlation between BAI scores and pause duration in both E-C (*r* = 0.926, *p* < 0.01) and C-E tasks (*r* = 0.951, *p* < 0.01).

## Discussion

### Translation anxiety and cognitive load

As language is of embodiment in nature and to a great extent, translation can be regarded as a kind of embodied-cognitive activity (Wang, 2021). That is why translation process is a sort of cognitive process, which takes cognitive load (total amount of cognitive resources consumed by human information processing) as an index to explain the cognitive processing of translators. To accomplish a task, an individual uses the limited cognitive-psychological resources in working memory at which point cognitive load transpires (Sweller, 1988).

The results of the present study suggest that the FLA in translation correlates positively with the cognitive load. The students performing both E-C and C-E translation tasks with higher cognitive load attained higher BAI scores. The findings offer support for Eysenck and Calvo (1992) PET theory which argues that cognitive efficiency is likely to suffer as stress or anxiety increases. As highlighted by Eysenck and Calvo (1992), intrusive thoughts irrelevant to tasks engage working memory capacity and hinder the efficiency of the cognitive process. The divided attention is likely to cause the decrease of the capacity demanded by information processing. As a result of the decrease in cognitive efficiency, it is likely to take longer to achieve good performance, otherwise, the performance is likely to become worse. The effect is noticed more easily when tasks are challenging or carried out under a high cognitive load (Baddeley and Hitch, 1974; Osborne, 2006; Derakshan and Eysenck, 2009). The findings also provide some support for Arnold and Brown (1999) claims that learners’ cognitive activities are likely to stop automatically when they are in a negative emotional state, and that only by using emotion and cognition at the same time can the learning process be built on a firmer foundation.

Furthermore, the results align with the findings of the study carried out by Chen et al. (2009). Chen reported some evidence in support of the existence of a relationship between FLA and cognitive load in the context of English listening comprehension. The current study contributes more empirically-substantiated findings in relation to the link between FLA and cognitive load, and offers some evidence for the existence of positive relationship between cognitive load and FLA in the context of L1 and L2 translations.

The results of this experiment not only provide stronger substantiation of the PET model which depicts the relationship between anxiety and cognitive load uni-directionally without depicting the effects of cognitive load on anxiety, i.e., it only examined the influence of anxiety on cognitive load, but also set up the relationship between anxiety and cognitive load bi-directionally. In other words, the results indicate that cognitive load is positively correlated with anxiety and that the increase of cognitive load is likely to lead to an increase in anxiety.

**Figure 7:**
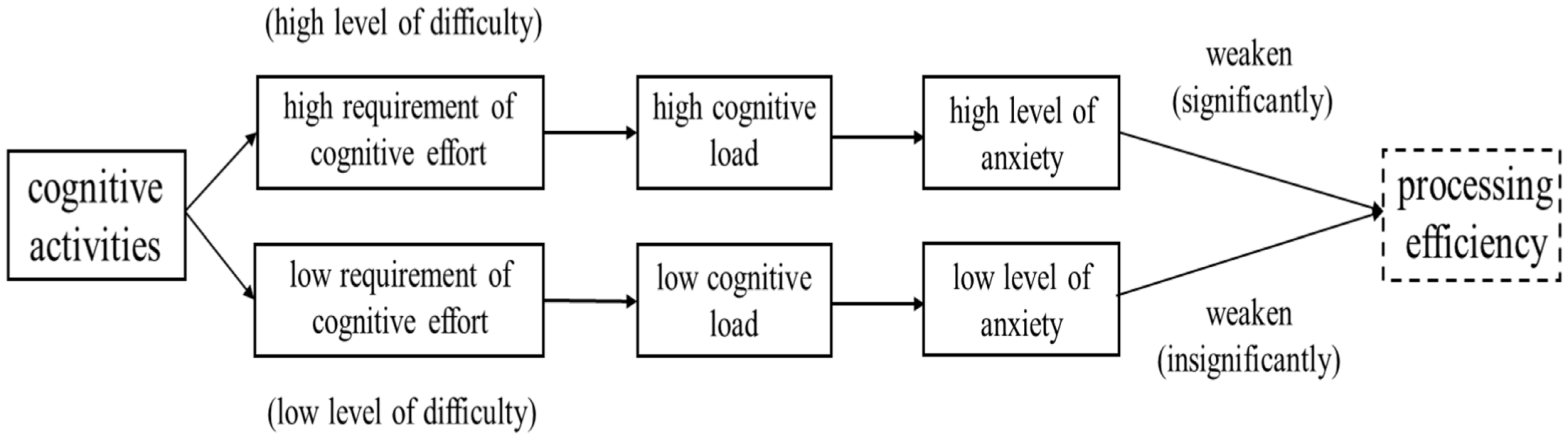
The correlation between cognitive load and anxiety.

Compared with the previous studies that they only investigated the relationship between anxiety and cognitive load by grouping subjects according to anxiety evaluation prior to experimentation, the present study was designed to solve the limitations of previous studies. To ensure that any anxiety identified in the participants could be clearly linked to the cognitive load of the directionality of the translation tasks rather than pre-existing affective states, the experiment in the present study assigned participants to two groups randomly, set them up to complete two translation tasks and then evaluated their levels of anxiety. Such a structuring of design allowed a clearer understanding of the antecedents of the participants’ anxiety. The findings of this study can be clearly observed in Figure 7.

As Figure 7 shows, the level of anxiety is determined by the degree of cognitive effort involved which in turn produces a corresponding level of cognitive load in cognitive activities. This implies that the attendant anxiety will attenuate the individual’s processing efficiency and lead to a low cognitive performance.

### More anxiety in L2 translation than in L1 translation

The results show that the BAI scores of the participants undertaking the L1 translation are significantly lower than those identifiable in the L2 translation task. This establishes that more anxiety is experienced in the process of L2 translation. In L1 translation, the participants’ BAI scores range from 1 to 11 with an average of 5.61 points, while the participants’ BAI scores in L2 translation range from 3 to 14 with an average of 7.80 points. With reference to the relationship between anxiety and translation direction, there is a significant difference of BAI scores between L1 and L2 translation tasks, i.e., the BAI scores of the participants in L1 translation is significantly lower than that in L2 translation. The difference can be explained by the correlation between cognitive load and anxiety.

For assessing cognitive load in L1 and L2 translations, variables such as task duration, fixation count, fixation duration, pause count and pause duration collected in the E-C and C-E translation tasks were compared in this study. Based on the results, the study has found that the value of these five variables of the participants in L2 translation are significantly higher than those in L1 translation, suggesting higher cognitive load and processing difficulty in L2 translation. This finding is congruent with the results reported in some previous studies. Feng (2017) compared the cognitive load of 20 student translators in L1 and L2 translations based on their eye-tracking data during translation processes and found that the cognitive load during L2 translation was higher than the cognitive load experienced during L1 translation.

In order to explore the reason of such a difference, a comparison between fixation time and reading time on the ST and TT in both translation directions was conducted in this study. According to the results and analysis, both fixation count and reading time on the ST in L1 translation is higher than that in L2 translation, though the difference is not significant. In the case of cognitive load in the target-text processing, both fixation count and reading time on the TT in L1 translation is lower than that in L2 translation, which means that the cognitive load placed on the TT (Chinese) in L1 translation is lower than that on the TT (English) in L2 translation. It can be found from the results presented above that the participant’s cognitive load in English processing is higher than that in Chinese processing in the process of translation. We may find some support from previous studies. Jensen and Pavlović (2009) used eye-tracking technology to investigate the distribution of translators’ cognitive load in L1 and L2 translations. They tried to test whether the cognitive effort invested in the processing of the ST was higher in L1 translation than that in L2 translation, and whether the cognitive effort invested in the processing of the TT was higher in L2 translation than that in L1 translation. Their results only confirmed their expectation that the TT processing requires more cognitive effort than the ST processing in both directions of translation. However, the results of the present study suggest that there may exist a “L2 effect” in translation, in which the second or foreign language usually receives more attention than the native language irrespective of translation direction.

Furthermore, owing to the less significant difference of cognitive load in the ST processing than that in the TT processing between L1 and L2 translations, the language type of the TT tends to have a greater impact on the translator’s cognitive load in the process of translation. The translation process can be seen as the combination of language comprehension and language output. The translator needs to get access to the concepts of ST and then output it with another language. High-level language learners do not need too much cognitive load for both first language comprehension and second language comprehension since they have already achieved high English proficiency. Therefore, certain second language words are directly linked to the concepts in a person’s mind instead of associating to the first language words.

The findings above also offer support for the RHM model. According to this model, high-level learners can directly access the conceptual system from second language vocabulary, while low-level learners access the conceptual information of second language vocabulary depending more on the first language translation, which indicates that it takes more cognitive load to understand the concepts of second language for low-level learners. In the present study, all the participants have passed CET-4 with the average score of 572.1, and based on their English proficiency, they can be regarded as medium- or high-level learners. Therefore, it was not very difficult for them to directly get access to the concepts of ST no matter it is L1 or L2, which helps to explain the insignificant difference of cognitive load between L1 and L2 in the source-text processing.

In the light of the above analysis, it is clear that the level of anxiety is closely related to the task difficulty, which is in turn reflected by cognitive load. For translators, L2 translation is more difficult than L1 translation due to the higher cognitive load involved in the former. The translators’ understanding and output of their mother tongue is a natural process, while the understanding and output of the second or foreign language is affected by many other external factors, such as social culture and language environment which leads to an increasing complexity for L2 translation.

### Cognitive mechanism of anxiety in L1 and L2 translations

**Figure 8:**
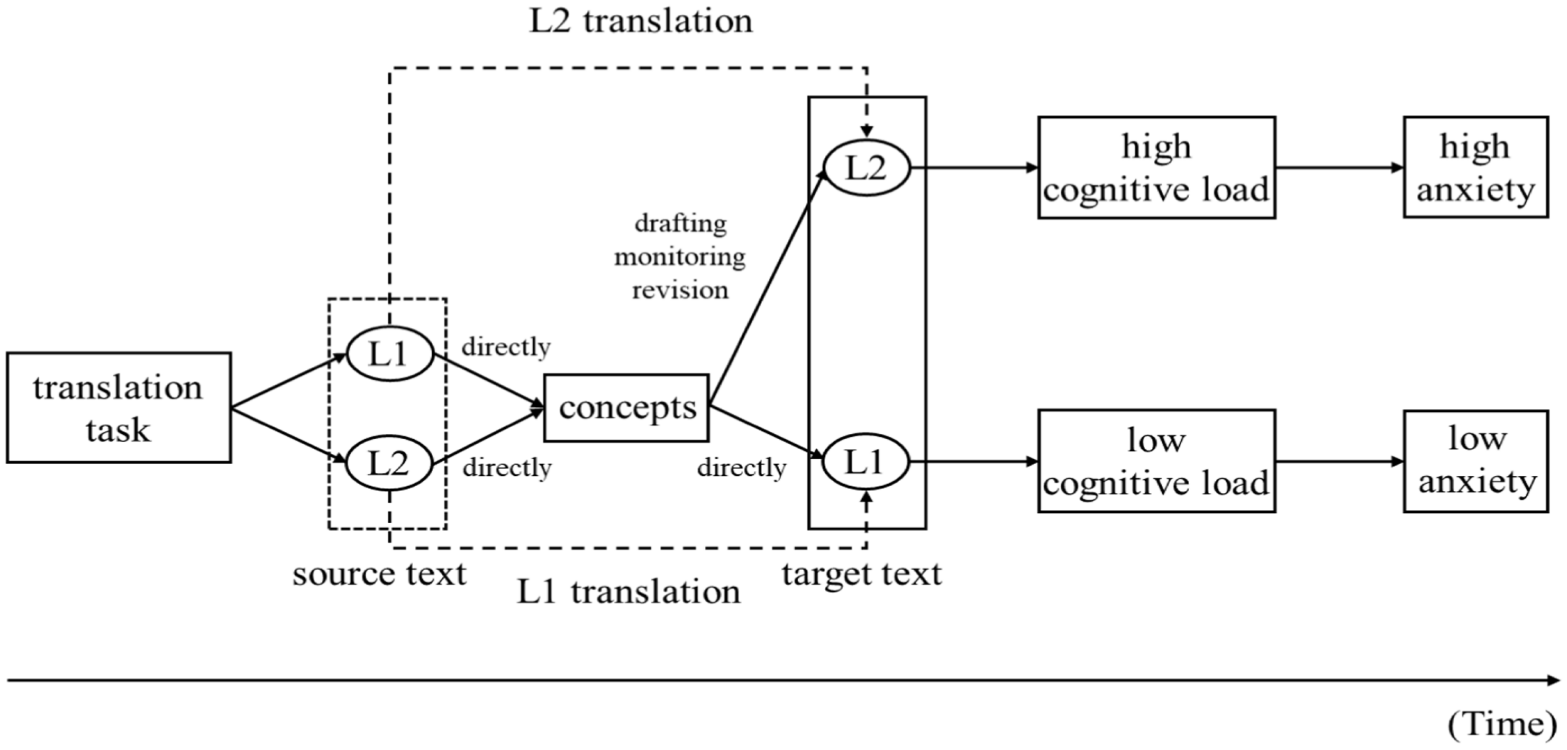
The cognitive mechanism of anxiety in L1-L2 translations.

Based on the data we collected from the translation anxiety experiment and the above discussion, a general cognitive mechanism model of translation anxiety under the influence of translation direction is presented in Figure 8. It delineates the timeline from the time of the translators’ first encounter with the translation task to the time of their translation production. From this model, we can see the different anxiety levels of translators instantiating between L1 translation and L2 translation.

The reason why the translator feels anxious when a L2 translation is provided can be explained with reference to the complexity of translation task (Wang and Wang, 2018). L2 translation is more difficult than L1 translation, because it requires more efforts for processing (e.g., monitoring and revision) which generates greater cognitive load and gives rise to higher anxiety. As the model shows, when a medium or high-level proficiency translator receives a translation task, he/she initially processes the ST in L1 or in L2 translation from the early period to the intermediate period in a nearly similar way. A high-level language learner is able to get access to the concepts directly no matter the ST is given in the first language or second language. During the early period, if the ST is presented in the first language, it is easy for the translator to understand his/her mother tongue and get the concepts. If the translator encounters the ST in the second language, due to his/her solid grasp of the second language knowledge, he/she does not need much cognitive efforts in the process of language comprehension.

After the concepts are obtained, the language type of TT determines the level of cognitive load in the processing which follows. In the stage of language output, L2 translation requires more cognitive efforts compared to L1 translation as the translator needs to do drafting, monitoring and revision before producing the TT. Such extensive cognitive efforts may result in a higher level of cognitive load due to the complicated nature of cognitive activities in the process of target language output, which in turn impacts the anxiety level of the translator.

The reason for higher anxiety in L2 translation can also be explained with reference to the translator’s previous experience of language learning and translation practice. In later translation tasks, after confirming the translation direction, the translator’s anxiety will be released in advance according to the previous experience (experience of task and memory of anxiety), thus giving rise to anticipatory anxiety prior to the commencement of the translation task.

## Conclusion

The study has found that cognitive load and anxiety in the process of translation are positively correlated, thus suggesting that a high cognitive load is likely to give rise to high anxiety in translation. It has also revealed that there were significant differences between task duration, fixation count, fixation duration, pause count and pause duration discernible in the two translation direction tasks. Further, the average value of the five variables in the C-E task (L2 translation) was significantly higher than that identified in the E-C task (L1 translation), thus implying that cognitive load in L2 translation is significantly higher than in L1 translation.

The difference of cognitive load between the two translation tasks is found to be attributable to the difficulty of the two translation tasks. For medium- or high-level language learners, L1 translation has proved to be less difficult than L2 translation. This is possibly because irrespective of the language of ST (mother tongue, second language or foreign language), language comprehension tends to be semi-automatic without requiring excessive cognitive effort. However, in the process of language output, the production of mother tongue is notably easier than the production of second or foreign language, thus reflecting the difference in difficulty between L1 and L2 translations. Meanwhile, the higher BAI scores observed in participants performing L2 translation also suggest that in comparison with L1 translation, L2 translation entails greater anxiety. Therefore, it would appear that L2 translation is likely to be more difficult for translators than L1 translation and lead to greater translation anxiety.

In relation to the third question, the study found that the cognitive load of processing in L2 was greater than that for L1 across L1 and L2 translations. The analysis of data showed that it was the language type of TT rather than ST which contributed to the cognitive load in the subsequent processing procedure. In summary, the results of the study corroborate the key premises of the Processing Efficiency Model and the REM, confirming that the taking up of working memory resources by anxiety negatively impacts the processing efficiency and that high-level learners can directly access the conceptual system from the second language vocabulary rather than through the first language vocabulary. The current study also offers important insights in relation to the relationship between translation directionality and anxiety. As a small-scale but original contribution to the body of knowledge, the current study further delineates the cognitive mechanism of L2 translation task performance and anxiety.

To test whether the findings of this research hold true, future research may be designed with a larger sample size of subjects and/or investigate educational settings, unlike the present setting (a top university in China) reputed for being more achievement-oriented (and thus likely to host learners subject to greater performance anxiety) than locales elsewhere. The practical implications of this study pertain to translation pedagogy in particular. Translation teachers should work to increase L2 translation practice so as to improve their students’ familiarity with the translation tasks and reduce attendant anxiety as well as encourage their students by giving positive feedback during translation practice in order to build up their confidence and decrease their fear of L2 translation.

## Data availability statement

The raw data supporting the conclusions of this article will be made available by the authors, without undue reservation.

## Ethics statement

The studies involving human participants were reviewed and approved by Research Ethics Board of Zhejing University. The patients/participants provided their written informed consent to participate in this study.

## Author contributions

ZW and XW conceived and designed the experiments. ZW and HC performed the experiments. JJ, ZW, and XW analyzed the data and wrote the manuscript. JJ and XW revised the manuscript. All authors contributed to the article and approved the submitted version.

## Conflict of interest

The authors declare that the research was conducted in the absence of any commercial or financial relationships that could be construed as a potential conflict of interest.

## Publisher’s note

All claims expressed in this article are solely those of the authors and do not necessarily represent those of their affiliated organizations, or those of the publisher, the editors and the reviewers. Any product that may be evaluated in this article, or claim that may be made by its manufacturer, is not guaranteed or endorsed by the publisher.

## Supplementary material

The Supplementary material for this article can be found online at: https://www.frontiersin.org/articles/10.3389/fpsyg.2023.1120140/full#supplementary-material

## Footnotes

^1^CET-4 refers to the Chinese College English Test Band 4, which is an important benchmark to test the college students’ English proficiency in China.
