# Supplementary material for: Translation directionality and translator anxiety: Evidence from eye movements in L1-L2 translation
Source: Front Psychol. 2023 Feb 21;14:1120140. doi: 10.3389/fpsyg.2023.1120140 (PMC9989210; doi:10.3389/fpsyg.2023.1120140)
Supplement: Supplementary file 1 [file Data_Sheet_1.docx]

# Appendix S1 Pre-test Texts

**L1 translation (English to Chinese):**

Online shopping has become one of people's favorite ways of shopping. For consumers, online shopping offers not only convenience, broader selection and competitive pricing, but also easier access to goods information. For businesses, the Internet brings in more customers and offers a larger market. For the entire market-oriented economy, this new type of shopping method can allocate resources with greater efficiency on a broader scale in a more extensive dimension.

**L2 translation (Chinese to English):**

中国是目前世界上第二位能源生产国和消费国。能源供应持续增长,为经济社会发展提供了重要的支撑。中国已经成为世界能源市场不可或缺的重要组成部分,对维护全球能源安全,正在发挥着越来越重要的积极作用。
