# Supplementary material for: Translation directionality and translator anxiety: Evidence from eye movements in L1-L2 translation
Source: Front Psychol. 2023 Feb 21;14:1120140. doi: 10.3389/fpsyg.2023.1120140 (PMC9989210; doi:10.3389/fpsyg.2023.1120140)
Supplement: Supplementary file 2 [file Data_Sheet_2.docx]

# Appendix S2 Formal Texts

**L2 translation:**

旅游是一项集观光、娱乐、健身为一体的愉快而美好的活动。旅游业随着时代进步而不断发展。古往今来，旅游一直是人们增长知识、丰富阅历、强健体魄的美好追求。在古代，中国先哲们就提出了“观国之光”的思想，倡导“读万卷书，行万里路”，游历名山大川，承天地之灵气，接山水之精华。

20世纪中叶以来，现代旅游在世界范围内迅速兴起，旅游人数不断增加，旅游产业规模持续扩大，旅游经济地位提升，旅游活动日益成为各国人民交流文化、增进友谊、扩大交往的重要渠道，对人类活动和社会进步产生着越来越广泛的影响。

**L1 translation:**

Tourism represents a kind of popular and pleasant activity that combines sightseeing, recreation and outdoor exercise, and it has been developing with the progress of the times. From ancient times till now, tourism has demonstrated people's happy wish for more knowledge, varied experience and good health. In ancient times, ancient Chinese thinkers raised the idea of “appreciating the landscape through sightseeing”. Ancient people also proposed to “travel ten thousand li and read ten thousand books”, which shows they found pleasure in enriching themselves mentally and physically through traveling over famous mountains and rivers.

Since the middle of the 20th century, modern tourism has been growing at a fast pace around the world. The number of tourists has ever been on the rise, the scale of the tourism industry has been on constant expansion, and the position of tourism in the economy has been obviously raised. Tourism serves gradually as an important bridge of cultural exchange, friendship and further exchanges and is exerting more and more extensive influence on the human life and social progress among various countries.
